# Laminin-integrin a6b4 interaction activates notch signaling to facilitate bladder cancer development

**DOI:** 10.1186/s12885-022-09645-7

**Published:** 2022-05-18

**Authors:** Nan Hao, Daming Yang, Tianpei Liu, Shucheng Liu, Xinsheng Lu, Libo Chen

**Affiliations:** 1 Department of Urology, the People’s Hospital of Guangxi Zhuang Autonomous Region, Nanning, 530000 Guangxi China; 2 Department of Urology, the First Affiliated Hospital, Hengyang Medical School, University of South China, Hengyang, 421200 Hunan China

**Keywords:** Laminin, Notch signaling, Bladder cancer, Integrins

## Abstract

**Background:**

Laminins are high-molecular weight (400 ~ 900 kDa) proteins in extracellular matrix, which serve as major component of the basal lamina, and play a crucial role in promoting tumor cell migration. This study aimed at characterizing the role of laminin in promoting cancer development, and elucidating the mechanism of tumor progression driven by laminin-Notch signaling in bladder cancer.

**Methods:**

2D collagen/laminin culture system was established and CCK-8/transwell assay was conducted to evaluate the proliferation/migration ability of Biu-87 and MB49 cells cultured on 2D gels. Activation of integrins-Notch1 signaling was determined by western blotting. Orthotopic bladder cancer mice model was established to assess the therapeutic effects of Notch inhibitor.

**Results:**

Our study demonstrated that extracellular laminin can trigger tumor cell proliferation/migration through integrin α6β4/Notch1 signaling in bladder cancer. Inhibition of Telomere repeat-binding factor 3 (TRB3)/Jagged Canonical Notch Ligand 1 (JAG1) signaling suppressed Notch signals activation induced by laminin-integrin axis. In MB49 orthotopic bladder cancer mice model, Notch inhibitor SAHM1 efficiently improved tumor suppressive effects of chemotherapy and prolonged survival time of tumor-bearing mice.

**Conclusion:**

In conclusion, we show that, in bladder cancer, extracellular laminin induced the activation of Notch pathway through integrin α6β4/TRB3/JAG3, and disclosed a novel role of laminin in bladder cancer cells proliferation or migration.

**Supplementary Information:**

The online version contains supplementary material available at 10.1186/s12885-022-09645-7.

## Background

Bladder cancer is one of most common malignant carcinomas, with an estimated 549,000 diagnosed cases and 200,000 deaths worldwide in 2018 [1]. Among newly diagnosed cases, muscle invasive bladder cancer (MIBC) account for approximately 20%, while Non-muscle invasive bladder cancer NMIBC accounts for 60-70, and 15% to 20% of NMIBC would progress to muscle invasive bladder cancer (MIBC) [2]. MIBC invades the detrusor muscle and is more likely to spread to lymph nodes than NMIBC, leading to poorer prognosis [3]. In fact, tumor metastasis is an important cause of poor prognosis in bladder cancer [4]. And many patients with bladder cancer still suffered from a high risk of distant metastasis after radical surgical treatment [5]. Therefore, a better understanding of molecular mechanism underlying bladder cancer progression may provide potential targets for the diagnosis and treatment of bladder cancer.

Cancer metastasis involves process of loss of cell-cell/matrix adhesions, proteolysis, and angiogenesis [6]. Basement membrane (BM), one specialized extracellular matrice, that underlies epithelia and endothelia, appears to play a crucial role during metastatic progression [6, 7]. BM is a meshwork of laminin, type IV collagen, nidogen, and proteoglycans, that holds cells and tissues together [8]. Initial tumor cells usually contacted with extracellular elements through cell surface receptors, which specifically bind to BM or other components in extracellular matrix. The matrix can be broken down by hydrolytic enzymes secreted by tumor cells, thereby resulting in escape of neoplastic cells from its site of origin [9]. Laminins are the most important component of the BM. Laminins are large molecular weight glycoproteins constituted by three disulfide-linked polypeptides, the alpha (α), beta (β), and gamma (γ) chains [10]. Laminins are produced by multiple cells, including nearly all epithelial-, smooth muscle-, cardiac muscle-, nerve- and endothelial cells [11]. Previous studies demonstrated that laminin tightly correlated with the progression of malignant tumors. Notably, laminin-5 loss from BM was found to be associated with an increased death rate in bladder cancer patients [12]. *LAMC1* gene, encoding laminin subunit gamma 1 (LAMC1) protein, has been demonstrated as a potent biomarker for aggressive endometrial cancer [13]. In brain cancers, loss of cell-surface laminin anchoring promotes tumor growth and correlated with poor clinical outcomes [14]. Several signaling pathways have been demonstrated to contribute to the proliferation and migration of tumor cells, including TGF-β signaling, MAPK-RAS-RAF signaling, Notch and Wnt/β-catenin pathway [15–18]. Notably, extracellular laminin can activate a number of intracellular signaling pathways, such as PI3K/AKT, MAPK/ERK, and Rho GTPases, through receptor engagement [19–21]. And the mechanisms of laminin involvement in tumor development of several cancer types, including lung cancer, colorectal cancer, and head and neck squamous carcinomas, via related signaling have also been reported [22–24]. However, little has reported on the molecular mechanism of laminin-induced tumorigenesis and progression in bladder cancer.

In this study, we demonstrated that laminin promoted cell proliferation and migration in bladder cancer via integrin-dependent biomechanical signals. Meanwhile, we elucidated the underlying mechanism of laminin-induced bladder cancer progression, which was dependent on an integrin α6β4/TRB3/JAG1/Notch signaling pathway. More importantly, blockade of Notch signals restrained the metastatic potential of bladder cancer cells, which provided novel insight in clinical bladder cancer therapy.

## Materials and methods

### Cell culture and reagents

Human bladder cancer cell line Biu-87 was purchased from the American Type Culture Collection (ATCC, Manassas, USA). Mouse bladder cancer cell line MB49 was a gentle gift from Peking Union Medical Collage. All cancer cells were cultured in Dulbecco’s modified Eagle’s medium (DMEM, Gibco, MA, USA) containing 10% fetal bovine serum (FBS) (Gibco, MA, USA), and maintained at 37 °C in 5% CO_2_. HCPT was purchased from Sangon (Shanghai, China). Notch inhibitor SAHM1 (C_94_H_162_N_36_O_23_S) was purchased from MedChemExpress (MA, USA). Laminin-1 for cell culture was purchased from Sigma-Aldrich (L4544, MA, USA).

For 2D collagen (containing laminin or not) gels culture, type I collagen (Solarbio, Beijing, China) was diluted to 2.5 mg/ml with DMEM culture medium (containing 2 μg/ml laminin or not). Subsequently, 20 μl 1 M NaOH solution were subsequently added into 230 μl collagen solution. 250 μl of the collagen mixture was seeded into a 24-well plate and mixed thoroughly. After 37 °C incubation for 1 hour, cancer cells were seeded on top of the solid 2D collagen gels at a concentration of 1 × 10^4^ cells/well and maintained in DMEM culture medium containing 10% FBS.

### Clinical specimens

Human bladder tumor tissue sections were obtained from the First Affiliated Hospital, University of South China, and divided into NMIBC and MIBC according to the Guidelines for the Diagnosis and Treatment of Bladder Cancer (2019). All participants and/or thier legal guardians agreed to participate in the study and informed in prior. The clinical experiments were carried out according to the Declaration of Helsinki. This study was approved by the Ethics Committee of the First Affiliated Hospital of University of South China (#20170257). Survival information of 405 bladder cancer patients in The Cancer Genome Atlas Program (TCGA) was downloaded from https://www.cbioportal.org.

### Cell proliferation assay

Cell proliferation was assessed by Cell Counting Kit-8 (CCK-8, Solarbio, Beijing, China). Briefly, MB49 or Biu-87 cells were seeded in 96-well plates (2500 per well) and cultured with DMEM culture medium supplemented with 10% FBS. Cell proliferation was examined at 0, 24, 48, and 72 hours according to manufacturer’s specifications. Absorbance of samples was quantified at 450 nm by microplate reader (Thermo Fisher, MA, USA). Cell proliferation was normalized to day 0 (2500 cells).

### Transwell assay

5 × 10^4^ MB49 or Biu-87 cells were seeded in the 8 μm transwell insert (Corning, CA, USA) containing 100 μl culture medium (10% FBS). The bottom chamber was filled with 500 μl culture medium containing 20% FBS. After 24 hours, the migrating cells were fixed with paraformaldehyde and stained with crystal violet. The migrating cells numbers were counted under an optical microscope (Leica, Munich, Germany).

### RNA interference

SiRNA to *ITGB4* (#siRNA1: 5′-GGUCACCUCCAAGAUGUUC-3; #siRNA2: 5′-GGACUGGGUCCUUUCACAU-3′), *ITGA6* (#siRNA1: 5′-GTGGGAAGTTTAATAGAGT-3; #siRNA2: 5′-CCTAGTGGGATATGCCTCCAGGTTA-3′), *JAG1* (#siRNA1: 5′-GGAACAACCUGUAACAUAGCCCGAA-3; #siRNA2: 5′-CCACAGCAACGAUCACAAAUGACTT-3′), *TRB3* (#siRNA1: 5′-CTTCGTCCAGCCCCAGTCC-3; #siRNA2: 5′-ATCTCTGGCTGCTTCTGCCGATGTT-3′) were purchased from Ruibo (Guangzhou, China). Transfections were performed in 24-well palates with 100 pmol siRNA and Invitrogen Lipofectamine 2000 (Thermo Fisher, MA, USA) according to manufacturer’s specifications. Quantitative Polymerase Chain Reaction (qPCR) was performed to examine the silence efficiency in Biu-87 cells.

### Quantitative polymerase chain reaction

MB49 or Biu-87 were cultured on dish or 2D laminin/collagen gels for 5 days. Cells were then harvested and total RNA was extracted using RNA Extraction Kit (Thermo Fisher, MA, USA) according to the manufacturer’s instructions. Reverse transcription of total RNA was performed using cDNA synthesis kits (Takara Bio, Tokyo, Japan) following the manufacturer’s instructions. PCR was performed with SYBR Green Supermixes (Biorad, MA, USA). Primer sequences were downloaded from https://pga.mgh.harvard.edu/primerbank/.

### Western blotting

Cancer cells were lysed by RIPA buffer (25 mM Tris, pH 7.6, 150 mM NaCl, 1% NP40, 1% DOC, 0.1% SDS). Protein samples were resolved by SDS–polyacrylamide gel electrophoresis and blotted on polyvinylidene difluoride membranes. Some of the blots were cut prior to hybridisation with antibodies. Primary antibodies, including anti-integrin α6 (ab181551), anti-integrin β4 (ab133682), anti-Notch1 (ab52627, cleaved intracellular domain of Notch1), anti-JAG1 (ab109532), anti-TRB3 (ab137526) and anti-β-actin (ab8226) were purchased from Abcam (Cambridge, UK). Full blots containing markers were included in supplementary materials.

### Immunohistochemistry and immunofluorescence

Bladder tumor tissues were fixed in 10% formalin solution. The samples were processed, embedded in paraffin, and sectioned at 5 μm for immunohistochemical and immunofluorescence staining. Sections of tumor tissues were then dewaxed, rehydrated, quenched of endogenous peroxidase, blocked, and incubated with the primary antibody: anti-Laminin (ab11575, Abcam, Cambridge, UK), anti-integrin α6 (ab181551, Abcam, Cambridge, UK), anti-integrin β4 (ab133682, Abcam, Cambridge, UK) and anti-Notch1 (ab52627, Abcam, Cambridge, UK) at 4 °C overnight. Samples were then incubated with secondary antibodies and stained with hematoxylin/ 4′, 6-diamidino-2-phenylindole (DAPI). The intensity of protein expression was quantified by Image J 2.0 (N.J, USA) and Image-pro Plus 6.0 software (MA, USA). 10 fields were included in each sample. The mean of brown intensity in 10 fields were identified as the expression intensity in this sample. 15 samples from 15 patients were included in each group.

### Dual luciferase activity assay

Activation of Notch1 signaling in tumor cells were determined by luciferase reporter assay. MB49 or Biu-87 cells were seeded on dish or 2D laminin/collagen gels for 3 days, and then co-transfected with control/pGL3 vector containing firefly luciferase reporter gene and the 30 UTR of Notch1 gene (Yunzhou, Beijing, China) using lipofectamine 2000 (Invitrogen, MA, USA). 48 hours later, a luciferase assay kit (Promega, MA, USA) was used for luciferase activity assay.

### Orthotopic animal models

Female C57BL/6 mice (6–8 weeks old) were purchased from Huafukang (Beijing, China). To establish orthotopic bladder cancer model, 1 × 10^6^ MB49 cells in 100 μl PBS were intravesical instilled into the bladders of C57BL/6 mice by venous indwelling needles. On day 6 and 8, mice were treated with PBS, HCPT (0.5 mg/ml), SAHM1 (0.5 mg/ml) or combining treatment by intravesical instillation. On day 10, the occurrence of hematuresis was recorded (*n* = 10). On day 12, mice were sacrificed for tumor weight analysis (*n* = 6). The tumor weight was calculated according to the formula: tumor weight = total bladder weight – normal bladder weight (21 mg). Survival of tumor bearing mice was recorded on a daily basis (n = 6). All animal experiments of this study were approved by the Institutional Animal Care and Use Committee of University of South China (20150223-154). The animal studies were conducted in accordance with the Public Health Service Policy and complied with the WHO guidelines for the humane use and care of animals.

### Statistical analysis

Each experiment was performed for three independent times. Data were presented as the mean ± SEM and statistical significance was analyzed using GraphPad 7.0 software (L.J, USA). Statistical significance between groups was calculated by Student’s t test for two groups or by one-way ANOVA for more than two groups. Bonferroni analysis were further used for the post hoc test. The survival rates were analyzed by Kaplan–Meier survival analysis. The survival information of clinical bladder patients was downloaded from https://www.cbioportal.org/. *p < 0.05; **p < 0.01; ns, no significant difference.

## Results

### Laminin promoted cell proliferation and migration in bladder cancer

**Fig. 1:**
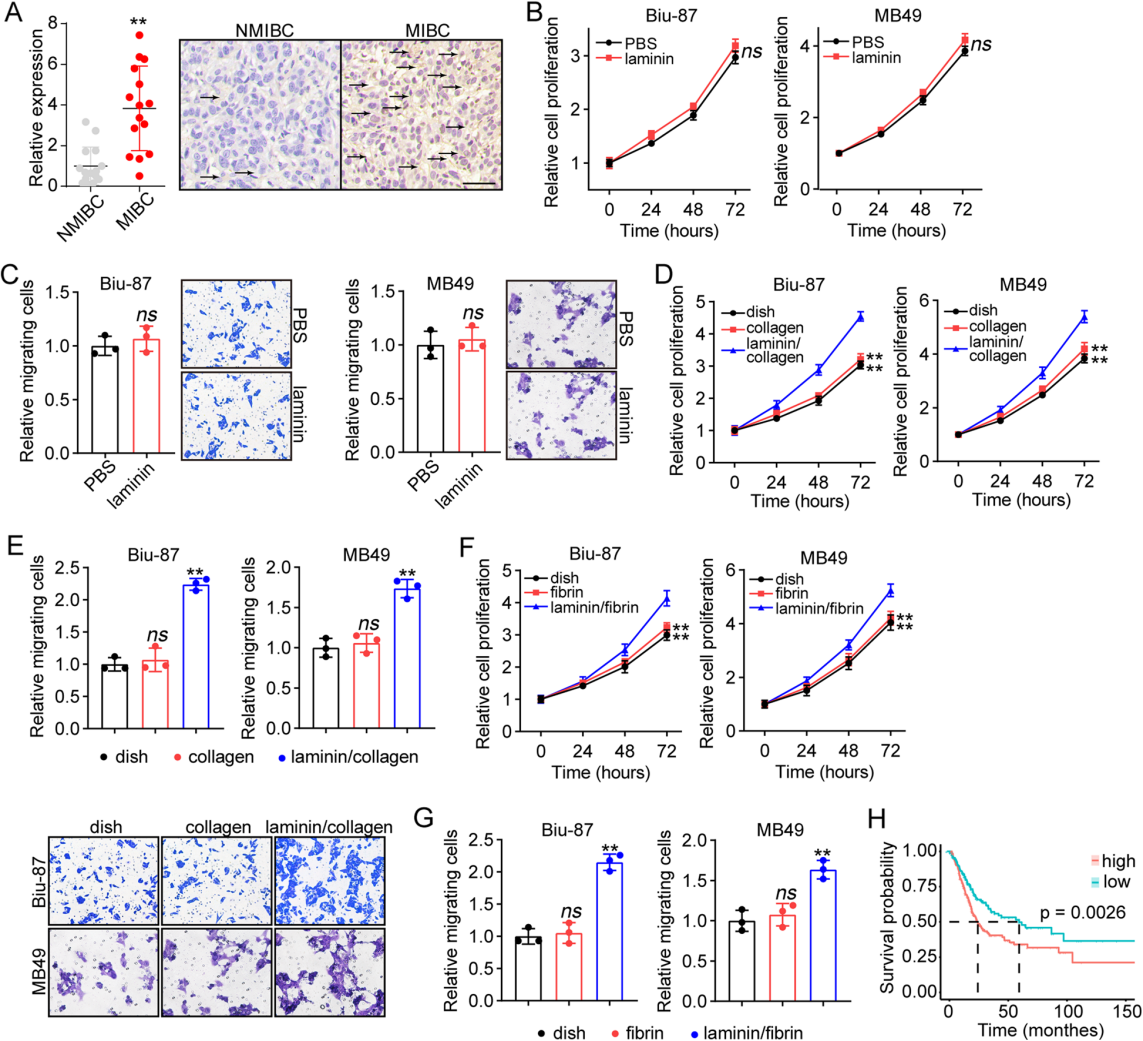
Laminin promoted cell proliferation and migration in bladder cancer. **A**, immunohistochemical staining of laminin in tumor tissues from NMIBC and MIBC patients. The expression of laminin was quantified in each group (*n =* 15). The scale bar was 50 μm. **B**, cell proliferation of Biu-87 and MB49 cells treated with PBS or laminin (2 μg/ml). **C**, the relative migrating cells of Biu-87 and MB49 cells treated with PBS or laminin (2 μg/ml). **D**, cell proliferation of Biu-87 and MB49 cells pre-cultured on dish, 2D collagen and 2D laminin/collagen gels. **E**, the relative migrating cells of Biu-87 and MB49 cells pre-cultured on dish, 2D collagen and 2D laminin/collagen gels. **F**, cell proliferation of Biu-87 cells pre-cultured on dish, 2D fibrin and 2D laminin/fibrin gels. **G**, the relative migrating cells of Biu-87 pre-cultured on dish, 2D fibrin and 2D laminin/fibrin gels. **H**, survival analysis of bladder cancer patients with high/low LAMC1 expression using TCGA database (*n =* 405)

To elucidate the role of extracellular matrix, more specifically, the role of laminin in tumor progression, laminin expression in bladder cancer patients was determined by immunohistochemistry. To do this, 30 patients were divided into NMIBC and MIBC groups according to the Guidelines for the Diagnosis and Treatment of Bladder Cancer (2019). As shown in Fig. 1A, tumor tissues from MIBC group exhibited a significantly increased laminin expression, when compared to the NMIBC group (Fig. 1A). This promoted us to speculate that laminin might play a role in bladder cancer development. To confirm our hypothesis, bladder cancer cell lines Biu-87 and MB49 cells were cultured with laminin for 3 days, and the cell proliferation/migration was determined by CCK-8/Transwell assay. However, no obvious difference was found in cell proliferation (Fig. 1B) or migration (Fig. 1C) between PBS and laminin treated group. As reported previously, extracellular laminin could contact with integrin receptors on tumor cells, promoting biomechanical signals transduction and activation of pro-survival signaling activation in tumor cells [19]. Based on this, we speculated that solid extracellular matrix-induced biomechanical force might play a role in laminin-integrin associated tumor progression. To assess our hypothesis, laminin was mixed in solid 2D collagen gels (type I collagen gels, the major extracellular substrate), then Biu-87 and MB49 cells were seeded on the top of 2D gels for cell culture. After 3 days, Biu-87 and MB49 cells were collected and cell proliferation/migration was determined. Intriguingly, 2D laminin/collagen complex culture significantly promoted Biu-87 and MB49 cells proliferation (Fig. 1D) and migration (Fig. 1E), whereas 2D collagen culture had limited impact on bladder cancer cells. Similar results were observed in laminin/fibrin 2D gels cultured cancer cells (Fig. 1F and G). Consistent to our results in vitro, a poor overall survival of bladder cancer patients with high LAMC1 (encoding laminin submit gamma 1) expression was observed by utilizing TCGA database analysis (Fig. 1H). Those results suggested that laminin could mediate the biomechanical signals transduction to promote tumor cells proliferation and migration, resulting in bladder cancer development.

### Laminin activated integrin α6β4 signals to promote tumor development

**Fig. 2:**
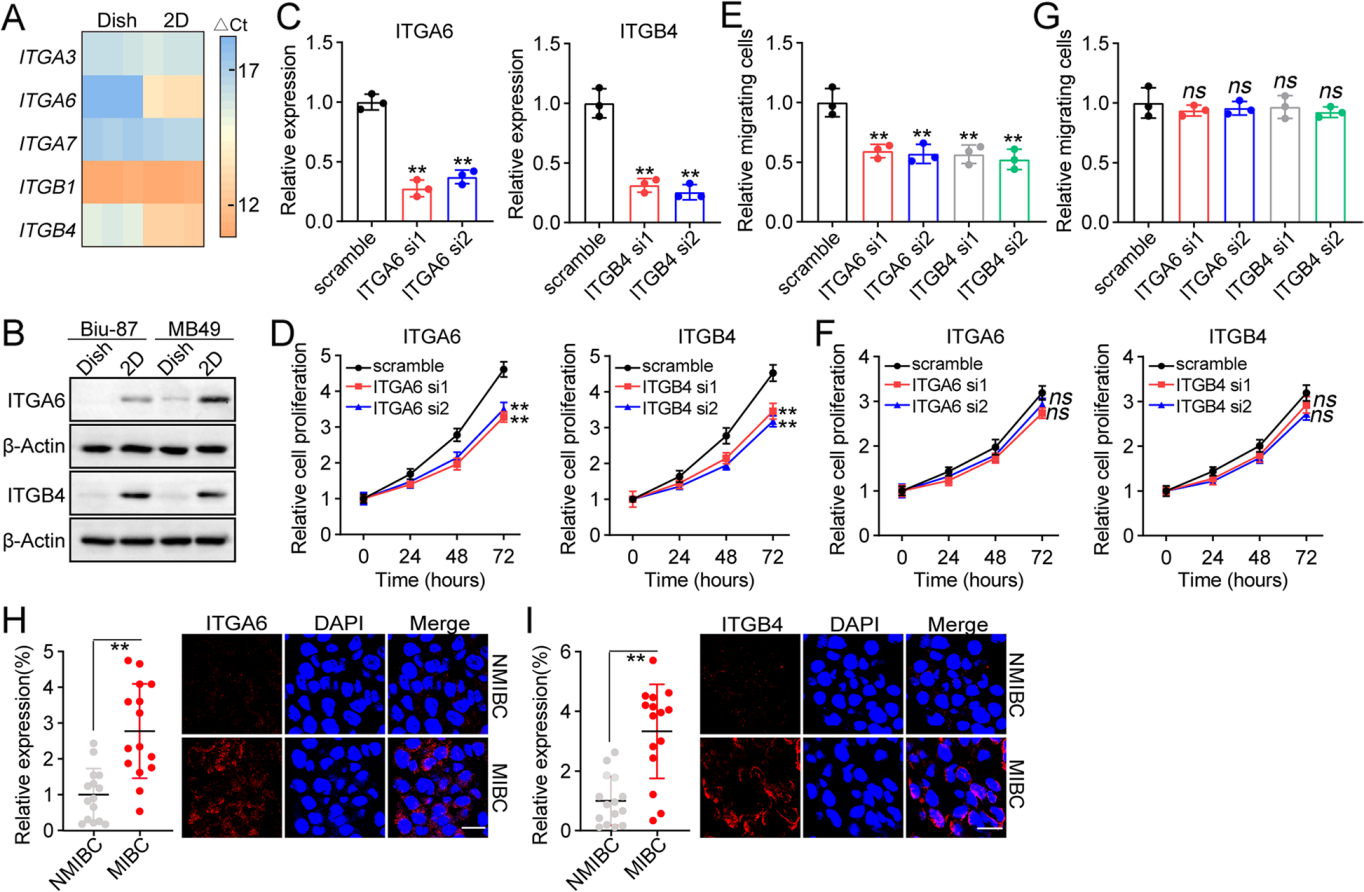
Laminin activated integrin α6β4 signals to promote tumor development. **A**, the expression of *ITGA3*, *ITGA6*, *ITGA7*, *ITGB1* and *ITGB4* in Biu-87 cells cultured on dish and 2D laminin/collagen gels. **B**, western blotting of integrin α6 and β4 in Biu-87 and MB49 cells cultured on dish and 2D laminin/collagen gels. **C**, the relative expression of *ITGA6* and *ITGB4* in Biu-87 cells treated with scrambled siRNA, ITGA6 siRNA and ITGB4 siRNA. **D**, cell proliferation of 2D laminin/collagen cultured Biu-87 cells treated with scrambled siRNA, ITGA6 siRNA and ITGB4 siRNA. **E**, relative migrating cells of 2D laminin/collagen cultured Biu-87 cells treated with scrambled siRNA, ITGA6 siRNA and ITGB4 siRNA. **F**, cell proliferation of dish cultured Biu-87 cells treated with scrambled siRNA, ITGA6 siRNA and ITGB4 siRNA. **G**, relative migrating cells of dish cultured Biu-87 cells treated with scrambled siRNA, ITGA6 siRNA and ITGB4 siRNA. **H**, immunofluorescence of integrin α6 in tumor tissues from NMIBC and MIBC patients. The expression of integrin α6 was quantified in each group (*n =* 15). The scale bar was 25 μm. **I**, immunofluorescence of integrin β4 in tumor tissues from NMIBC and MIBC patients. The expression of integrin β4 was quantified in each group (*n =* 15). The scale bar was 25 μm

We next sought to explore the underlying mechanism of laminin-associated tumor progression. As mentioned previously, laminin is recognized by integrin receptors, including integrin α3β1, α6β1, α7β1 and α6β4. And integrin signals-induced by laminin is tightly related to tumor growth and cancer metastasis. Here, we examined the expression of integrin α3, α6, α7, β1 and β4 in Biu-87 cells. Elevated expression of integrin α6 and β4 was observed in 2D laminin/collagen cultured Biu-87 cells by quantitative PCR (Fig. 2A). Similar results were observed at protein level in Biu-87 and MB49 cells (Fig. 2B), suggesting that integrin α6β4 might be involved in laminin-associated tumor progression. To further determine the role of integrin α6β4, integrin α6 and β4 were silenced by siRNA in Biu-87 cells (Fig. 2C), and cell proliferation/migration was determined. Accordingly, silence of integrin α6 or β4 suppressed the proliferative characteristics (Fig. 2D) and migrative phenotypes (Fig. 2E) in 2D laminin/collagen cultured Biu-87 cells. However, no obvious suppressive effects were observed in dish cultured cancer cells (Fig. 2F and G), despite integrin α6 or β4 siRNA treatment. Those results suggested that laminin promoted bladder cancer development through an integrin α6β4 dependent pathway. Next, we examined the expression of integrin α6 or β4 in tumor tissues from NMIBC and MIBC patients. Consistently, elevated expression of integrin α6 or β4 was observed in MIBC patients, when compared to the NMIBC group (Fig. 2H and I). Together, those results suggested that laminin activated integrin α6β4 signals to promote bladder cancer development.

### Integrin α6β4 promoted notch signals activation

**Fig. 3:**
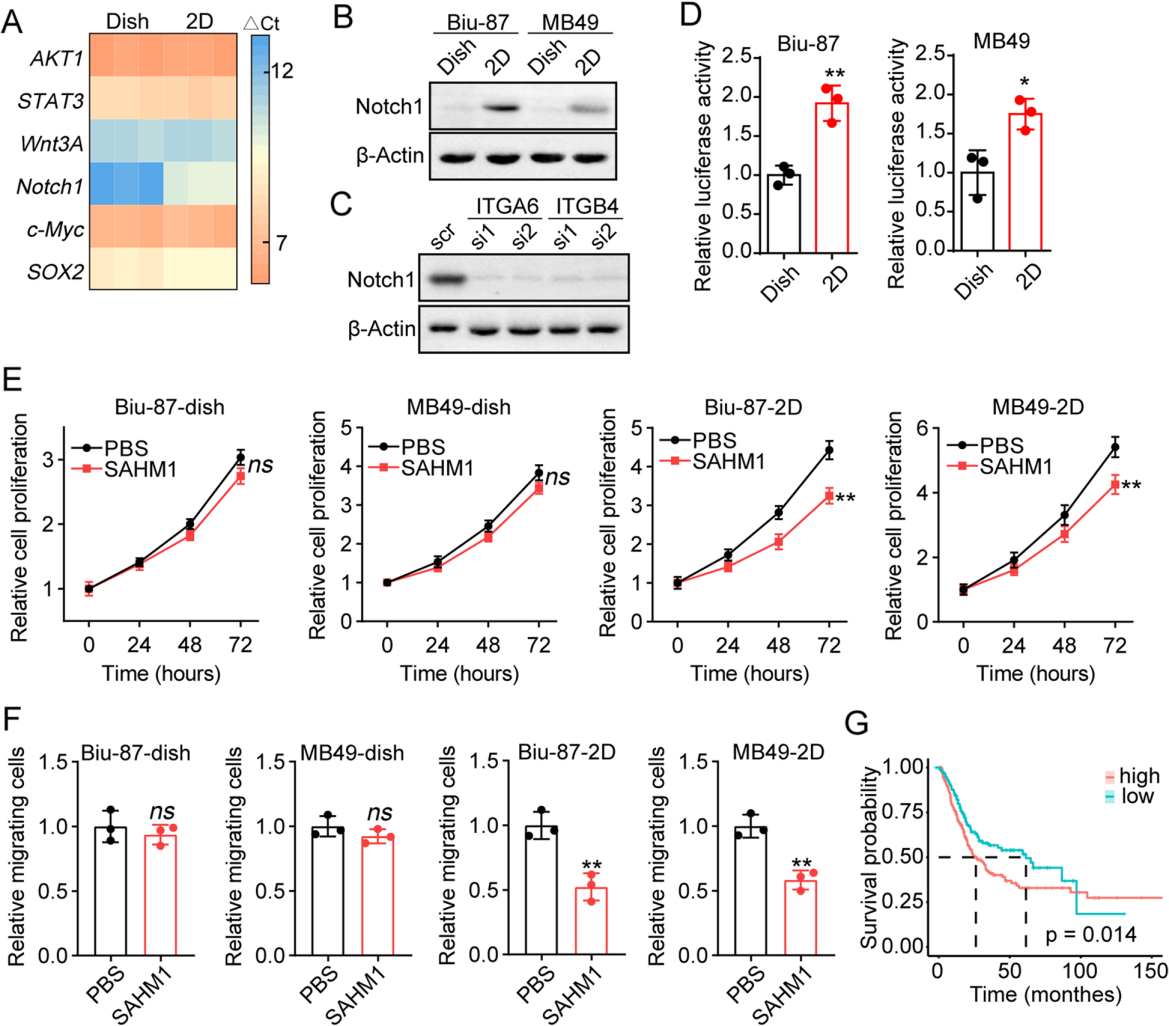
Integrin α6β4 promoted Notch signals activation. **A**, relative expression of *AKT1*, *STAT3*, *Wnt3A*, *Notch1*, *c-Myc* and *SOX2* in Biu-87 cells cultured on dish and 2D laminin/collagen gels. **B**, western blotting of cleaved intracellular domain of Notch1 in Biu-87 and MB49 cells cultured on dish or 2D laminin/collagen gels. **C**, western blotting of cleaved intracellular domain of Notch1 in Biu-87 cells cultured on 2D laminin/collagen gels (treated with scrambled siRNA, ITGA6 siRNA and ITGB4 siRNA). **D**, the full length human Notch1 promoter was cloned into the luciferase reporter vector. Notch1 transcription activity was examined after dish or 2D collagen/laminin culture (3 days) in Biu-87 and MB49 cells. **E**, cell proliferation of dish and 2D laminin/collagen cultured Biu-87/MB49 cells treated with PBS or SAHM1 (10 nM). **F**, relative migrating cells of dish and 2D laminin/collagen cultured Biu-87/MB49 cells treated with PBS or SAHM1 (10 nM). **G**, survival analysis of bladder cancer patients with high/low Notch1 expression using TCGA database (*n =* 405)

Compelling studies have demonstrated that integrins are involved in the activation of pro-survival signaling pathways, including PI3K/AKT, JAK/STAT3, Wnt, Notch, c-Myc and SOX2 signals. To clarify the mechanism of integrin α6β4-relating tumor progression, PCR analysis was performed to examine the expression of *AKT1, STAT3, Wnt3A, Notch1, c-Myc* and *SOX2* in 2D laminin/collagen or dish cultured Biu-87 cells. Intriguingly, the expression of *Notch1* was significantly upregulated in 2D laminin/collagen cultured groups (Fig. 3A). Additionally, 2D laminin/collagen cultured Biu-87 displayed an enhanced expression of cleaved intracellular domain of Notch1 at protein level, when compared to dish cultured group (Fig. 3B). Silence of integrin α6 or β4 suppressed the upregulation of Notch1 in laminin/collagen cultured cancer cells (Fig. 3C), suggesting that laminin promoted Notch1 signals activation through integrin α6β4 in bladder cancer. The above results were further confirmed by the luciferase assay by revealing that 2D collagen/laminin culture promoted Notch1 luciferase activity in MB49 and Biu-87 (Fig. 3D). To further confirm the role of Notch signaling in promoting bladder cancer development, a Notch inhibitor SAHM1 was added into the culture medium of tumor cells, and cell proliferation/migration was determined. Consistently, SAHM1 treatment obviously suppressed the cell proliferation (Fig. 3E) and migration (Fig. 3F) in 2D laminin/collagen cultured cells. However, limited tumor suppressive effects of SAHM1 were observed in dish cultured Biu-87 and MB49 cells, indicating that laminin promoted bladder cancer development through Notch-associated signaling. Importantly, a poor overall survival of bladder cancer patients with high Notch1 expression was observed by utilizing TCGA database analysis (Fig. 3G). Collectively, those results suggested that laminin upregulated integrin α6β4/Notch signaling to mediate bladder cancer development.

### The activation of notch was dependent on TRB3/JAG1 signaling

**Fig. 4:**
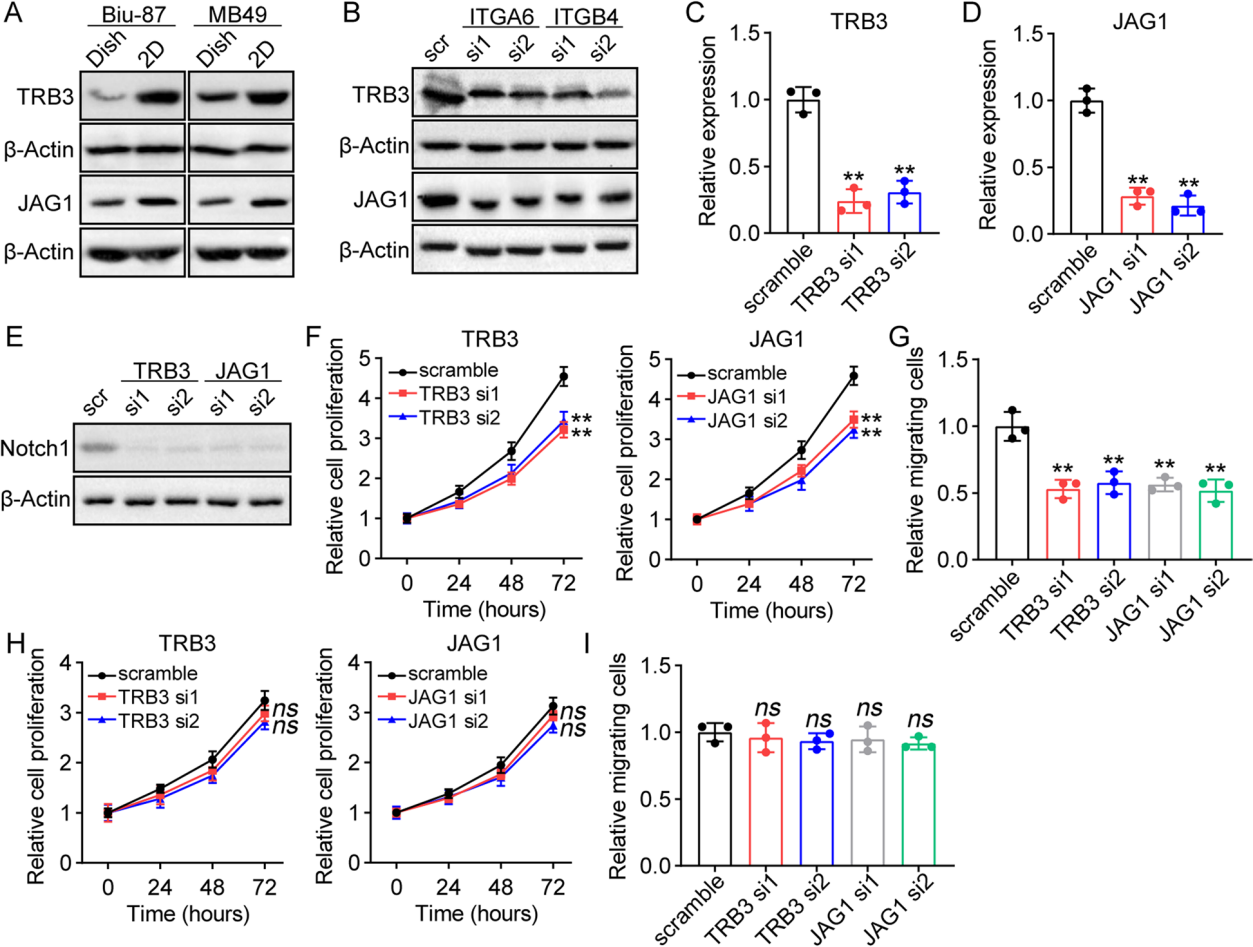
The activation of Notch was dependent on TRB3/JAG1 signaling. **A**, western blotting of TRB3 and JAG1 in dish and 2D laminin/collagen cultured Biu-87/MB49 cells. **B**, western blotting of TRB3 and JAG1 in 2D laminin/collagen cultured Biu-87 cells treated with scrambled siRNA, ITGA6 siRNA and ITGB4 siRNA. **C**, relative expression of *TRB3* in Biu-87 cells treated with scrambled siRNA and TRB3 siRNA. **D**, relative expression of *JAG1* in Biu-87 cells treated with scrambled siRNA and JAG1 siRNA. **E**, western blotting of Notch1 in 2D laminin/collagen cultured Biu-87 cells treated with scrambled siRNA, TRB3 siRNA and JAG1 siRNA. **F**, cell proliferation of 2D laminin/collagen cultured Biu-87 cells treated with scrambled siRNA, TRB3 siRNA and JAG1 siRNA. **G**, relative migrating cells of 2D laminin/collagen cultured Biu-87 cells treated with scrambled siRNA, TRB3 siRNA and JAG1 siRNA. **H**, cell proliferation of dish cultured Biu-87 cells treated with scrambled siRNA, TRB3 siRNA and JAG1 siRNA. **I**, relative migrating cells of dish cultured Biu-87 cells treated with scrambled siRNA, TRB3 siRNA and JAG1 siRNA

Last, we aimed to understand how integrin α6β4 controlled the activation of Notch signals in bladder cancer. Cellular stress has been reported previously to mediate the Notch signaling activation through TRB3/JAG1 axis [25]. Our results have demonstrated that laminin could mediate the biomechanical stress signals transduction through integrin α6β4, thereby promoting Notch signaling activation. Therefore, we presented that laminin/integrin α6β4 might facilitate Notch activation through TRB3/JAG1 signals. To confirm our hypothesis, western blotting analysis was performed to examine the expression of TRB3 and JAG1 on dish and 2D laminin/collagen cultured Biu-87/MB49 cells. Accordingly, cancer cells cultured on 2D laminin/collagen exhibited a higher expression of TRB3 and JAG1 (Fig. 4A), when compared to dish cultured groups. And silence of integrin α6 or β4 suppressed the upregulation of TRB3 and JAG1 (Fig. 4B), indicating that laminin upregulated TRB3/JAG1 through integrin α6β4. Subsequently, we silenced TRB3 and JAG1 in 2D laminin/collagen cultured Biu-87 by siRNA (Fig. 4C and D), then examined the expression of Notch1. Consistently, silence of TRB3 or JAD1 efficiently suppressed Notch1 in 2D laminin/collagen cultured Biu-87 (Fig. 4E), indicating that the activation of Notch was dependent on TRB3/JAD1 signals. Meanwhile, silence of TRB3 and JAD1 suppressed cell proliferation (Fig. 4F) and migration (Fig. 4G) induced by laminin, whereas limited suppressive effects were observed in dish cultured Biu-87 cells (Fig. 4H and I). Together, those results suggested that the activation of Notch in bladder cancer was dependent on TRB3/JAG1 signaling.

### Blockade of notch signals improved tumor suppressive effects in an orthotopic bladder cancer model

**Fig. 5:**
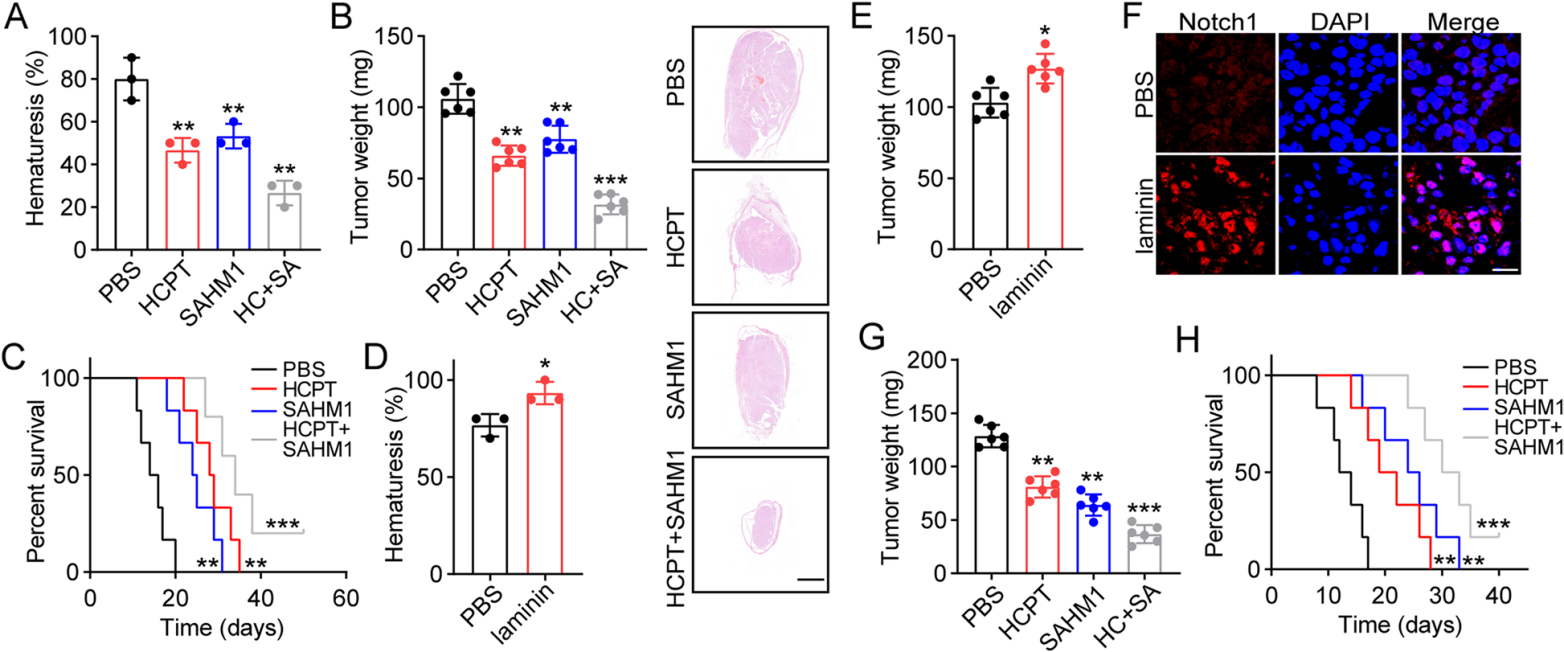
Blockade of Notch signals improved tumor suppressive effects in an orthotopic bladder cancer model. **A**, the rates of hematuresis (day 10) in tumor bearing mice treated with PBS, HCPT, SAHM1 or combining treatment (HC + SA). **B**, the tumor weight (day 12) of mice treated PBS, HCPT, SAHM1 or combining treatment (HC + SA). The H&E staining were presented and the scale bar was 2 mm. **C**, the overall survival of tumor bearing mice treated with PBS, HCPT, SAHM1 or combining treatment. **D**, the rates of hematuresis (day 10) in tumor bearing mice treated with PBS or laminin (1 μg per mouse). **E**, the tumor weight (day 12) of mice treated with PBS or laminin (1 μg per mouse). **F**, immunofluorescence of Notch1 in tumor tissues from (E). The scale bar was 25 μm. **G**, the tumor weight (day 12) of mice (laminin treatment) treated PBS, HCPT, SAHM1 or combining treatment (HC + SA). **H**, the overall survival of tumor bearing mice (laminin treatment) treated with PBS, HCPT, SAHM1 or combining treatment

Given the crucial role of laminin/integrin α6β4/TRB3/JAG1/Notch in promoting bladder cancer development, it could be feasible to suppress Notch signals for improved outcome in bladder cancer treatment. To validate our hypothesis, orthotopic bladder cancer model was established by instilling MB49 cells into bladders of C57BL/6 mice. Mice were treated with PBS, Notch inhibitor SAHM1, chemotherapeutic HCPT by intravesical instillation. Intriguingly, both HCPT and SAHM1 reduced hematuresis and suppressed tumor growth in MB49-bearing mice. Combination of Notch inhibitor and chemotherapy exhibited enhanced anticancer effects, which significantly inhibited tumor growth and prolonged the overall survival of tumor-bearing mice (Fig. 5A, B and C). Those results provided us new target to eliminate bladder cancer cells. Our previous results have pointed out that elevated expression laminin might promoted integrin/Notch signals activation, resulting sustained tumor growth in bladder cancer. Therefore, we treated those MB49-bearing mice with laminin by intravesical instillation, and further evaluated the anticancer effects of Notch inhibitors. Indeed, laminin treatment dramatically promoted bladder cancer development in vivo (Fig. 5D and E). Laminin treated tumor tissues also revealed enhanced expression of Notch1 in vivo (Fig. 5F). Next, we further treated those tumor-bearing mice (laminin treatment) with HCPT and SAHM1. Intriguingly, Notch inhibitor SAHM1 exhibited stronger anticancer effects when compared to HCPT, which might be associated with the chemo-resistance-induced by Notch associated signaling pathways. However, the combination of HCPT and SAHM1 displayed obvious tumor suppressive effects and prolonged overall survival of tumor bearing mice (Fig. 5G and H). Collectively, those results suggested that blockade of Notch signals improved tumor suppressive effects of chemotherapy.

## Discussion

Extracellular matrix is composed of various elements, such as collagen, proteoglycans, laminin, and fibronectin. Extracellular matrix plays a vital role in regulating crucial physiological processes, such as cell-cell communication, cell adhesion, and cell proliferation, which has been appreciated as important driver for cancer progression [26]. Previous studies on extracellular matrix in tumor progression mostly focused on collagen, fibronectin, and proteoglycans [27–29], while little study sheds light on the role of laminin. However, current studies revealed that laminin expression was tightly associated with tumor progression in several types of tumors. For example, laminin has been found to be involved in tumor invasion and metastasis in colorectal cancer, gastric cancer, and intrahepatic cholangiocarcinoma [30–32]. Our experiments revealed that laminin expression is significantly upregulated in human MIBC, which promoted us to hypothesize that laminin may play a role in bladder cancer. On this basis, we demonstrated that laminin could promote tumor cells proliferation and migration, leading to the development of bladder cancer. We firstly to confirm laminin, a major and important component of the ECM, contributes to the progression of bladder cancer.

Integrins are heterodimers consisting of one α subunit and one β subunit, which function as adhesion receptors for the ligands (e.g. laminin, collagen, and fibronectin) in extracellular matrix, which transduce mechanical signals from the extracellular matrix to stromal cells. Integrins α3β1, α6β1, α6β4 and α7β1 make up a laminin-binding integrins subfamily. The role of integrin α6β4 in promoting lung cancer, breast cancer, and colon carcinoma has been reported previously [33–35], however, there is little evidence for such role of α6β4 in bladder cancer. In this study, we indicated that laminin activated integrin α6β4 signals to promote bladder cancer development. We further explored the mechanism of laminin and integrin α6β4 in promoting bladder tumor progression. Much evidence has shown that integrin α6β4 is involved in the activation of pro-survival signaling pathways. For example, laminin-binding integrins induce Notch signaling in endothelial cells [36]. Additionally, integrin α6β4 promotes breast cancer cell motility and invasion through activating phosphatidylinositol-3-hydroxykinase signaling [37]. In lung cancer, activated integrin β4 recruits focal adhesion kinase to mediate downstream signaling pathways and cancer metastasis [38]. We demonstrated that laminin promoted Notch 1 signals activation through integrin α6β4, thereby facilitating bladder cancer cell proliferation and migration. In addition, the TCGA database analysis also revealed Notch 1 was associated with a poor prognosis of bladder cancer. Also, we found the activation of Notch 1 in bladder cancer is dependent on TRB3/JAG1 signaling. Taken together, we identified the relationship between laminin, integrin, Notch, and TRB3/JAG1 in bladder cancer.

Notch signaling has also been reported to be involved in the control of cell proliferation, survival, migration, and differentiation [39]. Intriguingly, the Notch pathway has been implicated in both oncogenic and tumor-suppressive roles in cancer depending on the tissue type and cellular context. In bladder cancer, Notch1 has also been reported to serve as tumor suppressive [40] and oncogenic roles [41] to regulate cancer cell proliferation and migration. Our study further demonstrated that laminin could mediate bladder cancer development through a Notch1 dependent manner. Intriguingly, our further investigation indicated that the pro-tumor effects of Notch1 might be tightly correlated with tumor-specific cell senescence and nutrition metabolism. Meanwhile, the laminin associated downstream signaling pathways might cooperate with Notch molecule, resulting in disparate tumor behaviors. The specific mechanism of Notch-induced tumor progression remains to be further investigated. Encouragingly, our experiments indicate that blocking Notch signals inhibited tumor growth and improved outcome of chemotherapy, which provided novel insight for bladder cancer therapy.

Based on the findings and limitations of previous studies, our study sheds further light on that laminin activated TRB3/JAG1/Notch signaling through integrin α6β4 to promote bladder cancer development. Firstly, our study identified that laminin, a major component of extracellular matrix, was significantly upregulated in patients with MIBC and demonstrated that laminin plays a critical role in bladder cancer development. Secondly, we indicated a novel signaling pathway in which laminin promotes tumor cells proliferation and migration via integrin α6β4/TRB3/JAG1/Notch axis. Thirdly, we elucidated the interrelationship between laminin, integrin, TRB3/JAG1, and Notch, which offered novel insight for tumor signaling pathways investigations. Fourthly, we proved that Notch inhibitor SAHM1 combining chemotherapeutic HCPT could inhibit tumor growth and improve prognosis, describing innovative strategy for the clinical treatment of bladder cancer. Finally, the novel signaling molecules, including laminin, integrin α6β4, and Notch1, can serve as potential prognostic and diagnostic indicators of bladder cancer.

## Conclusion

In summary, our study demonstrated novel mechanism of laminin-induced bladder cancer progression. The development of bladder cancer stimulated by the laminin/integrin α6β4/TRB3/JAG1/Notch pathway could be inhibited by Notch inhibitor SAHM1, which described a new strategy in the treatment of bladder cancer.

## Supplementary Information

**Additional file 1.**

## Data Availability

The anonymized data used and/or analyzed during the current study are available from the corresponding author on reasonable request.

## Abbreviations

MIBC: Muscle invasive bladder cancer
NMIBC: Non-muscle invasive bladder cancer
BM: Basement membrane
LAMC1: Laminin subunit gamma 1
TCGA: The Cancer Genome Atlas
TRB3: Telomere repeat-binding factor 3
JAG1: Jagged Canonical Notch Ligand 1
