# Supplementary material for: Laminin-integrin a6b4 interaction activates notch signaling to facilitate bladder cancer development
Source: BMC Cancer. 2022 May 18;22:558. doi: 10.1186/s12885-022-09645-7 (PMC9118635; doi:10.1186/s12885-022-09645-7)
Supplement: Supplementary file 1 — Additional file 1. [file 12885_2022_9645_MOESM1_ESM.docx]

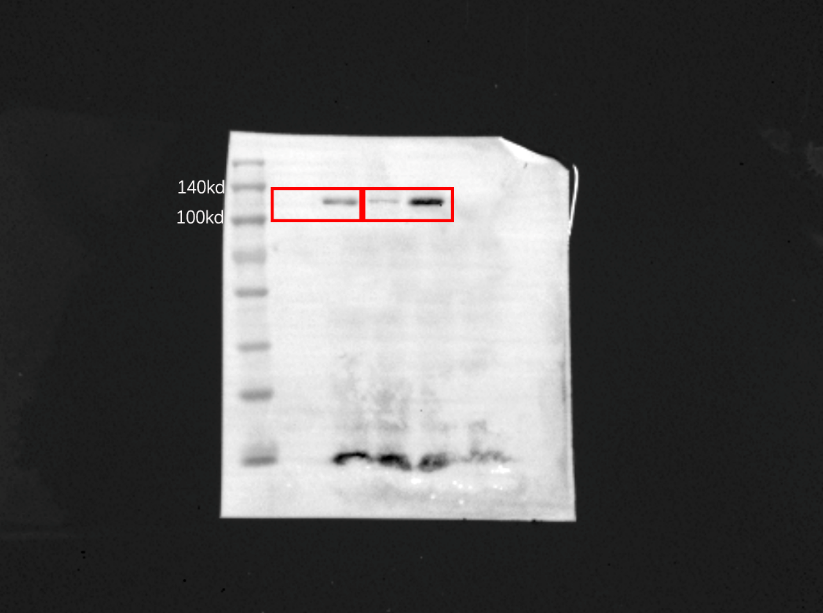


Fig2B ITGA6 (left-BIU-87 right-MB49)


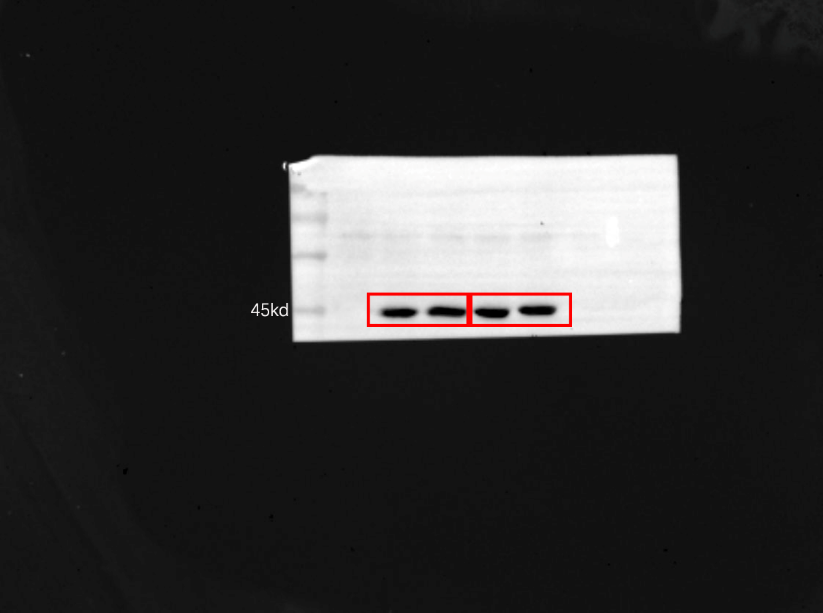


Fig2B Actin-1 (left-BIU-87 right-MB49)


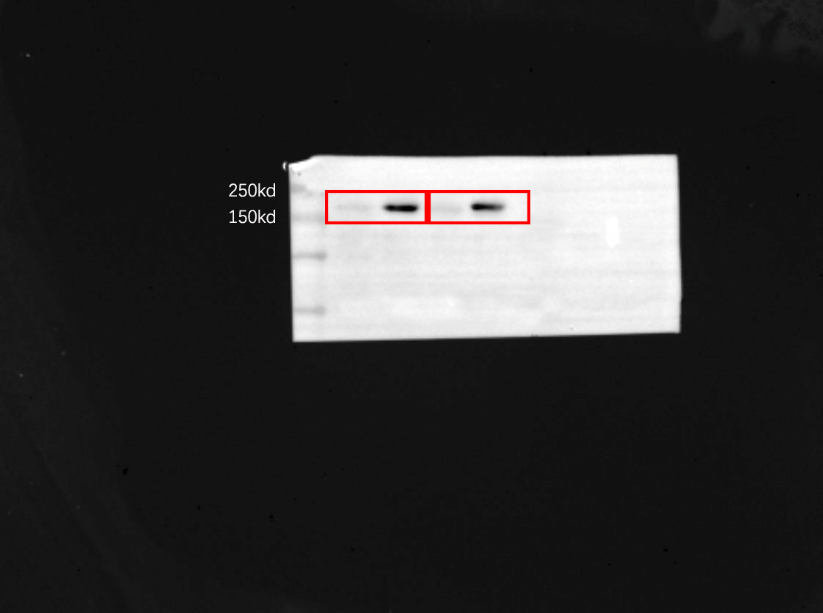


Fig2B ITGB4 (left-BIU-87 right-MB49)


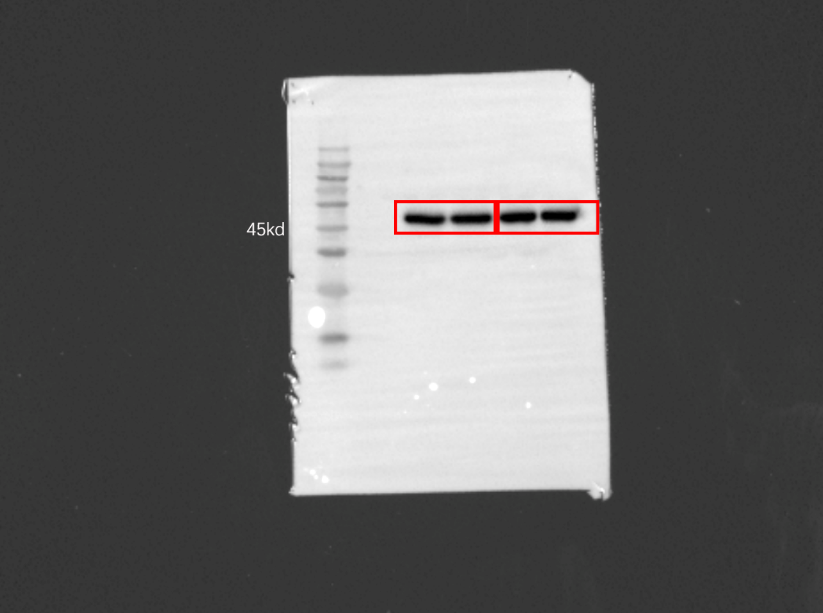


Fig2B Actin-2 (left-BIU-87 right-MB49)


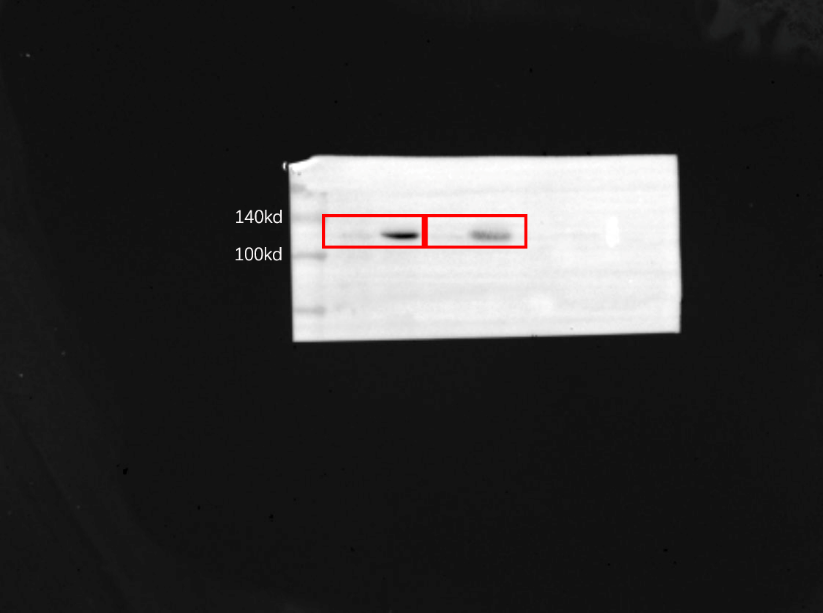


Fig3B Notch1 (left-BIU-87 right-MB49)
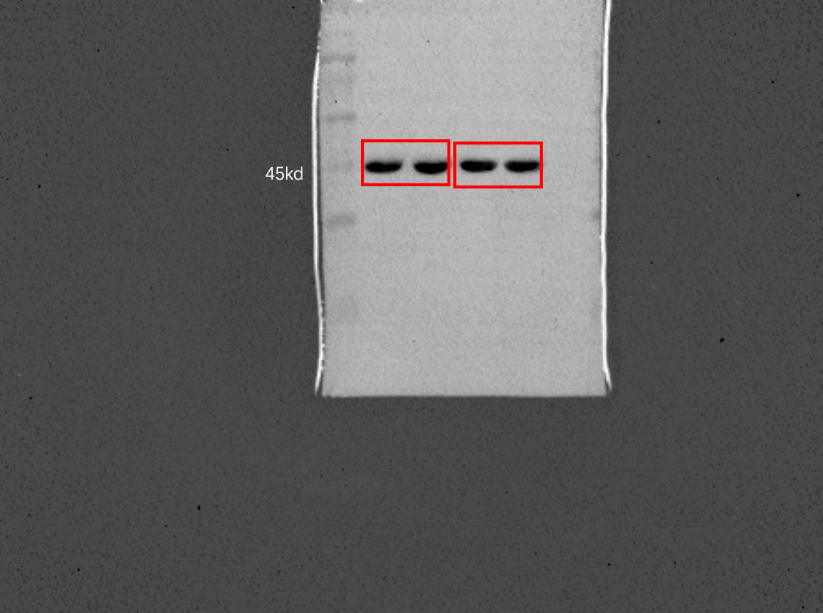


Fig3B Actin (left-BIU-87 right-MB49)


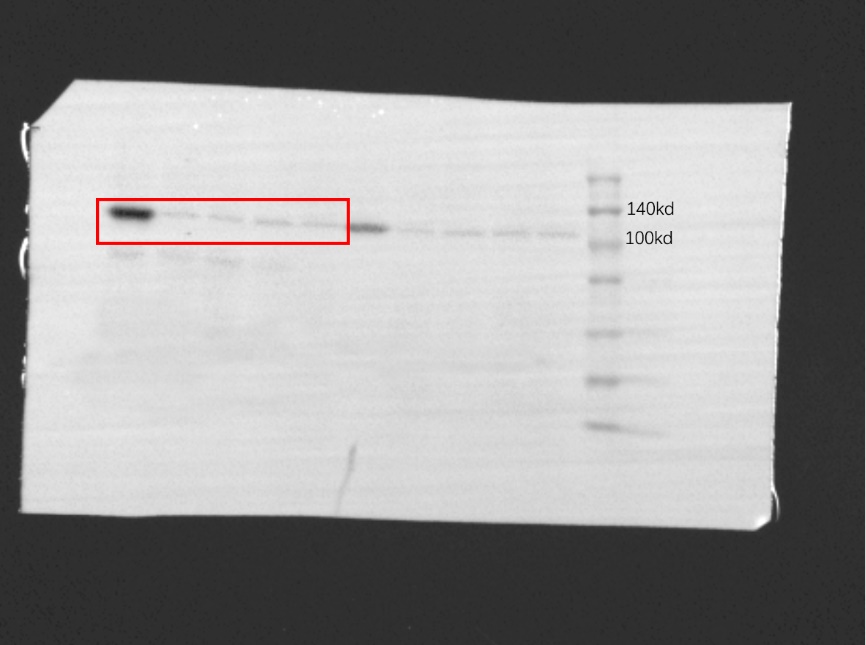


Fig3C Notch1


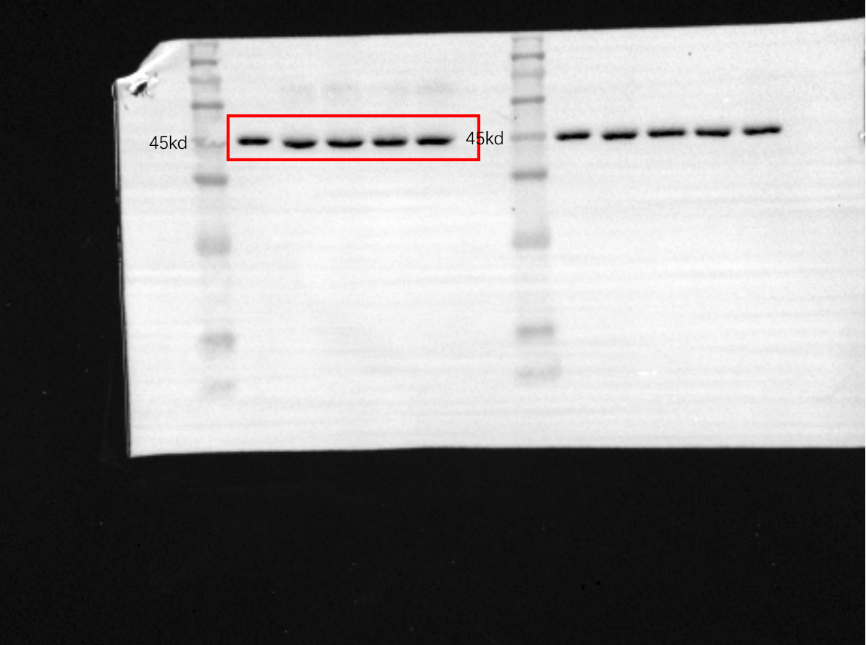


Fig3C Actin


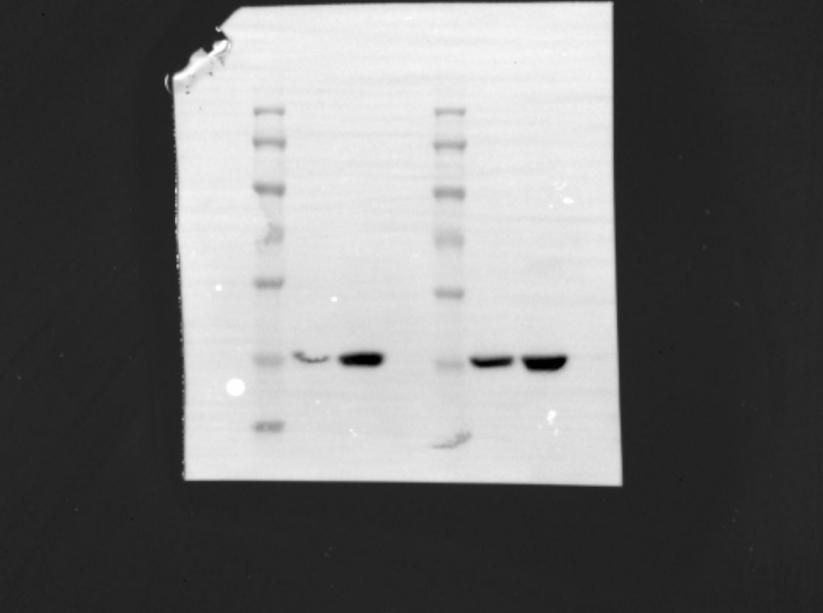


45kd

Fig4A TRB3 (left-BIU-87 right-MB49)


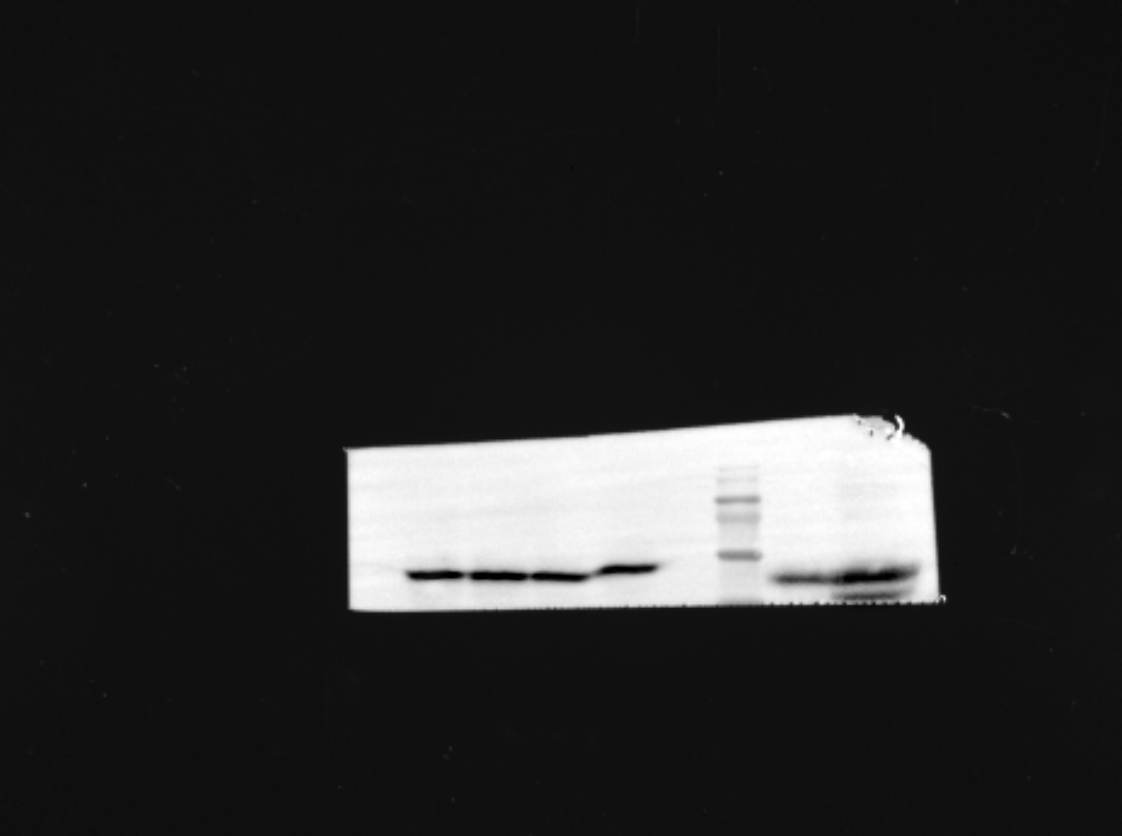


45kd

Fig4A Actin-1 (left-BIU-87 right-MB49)


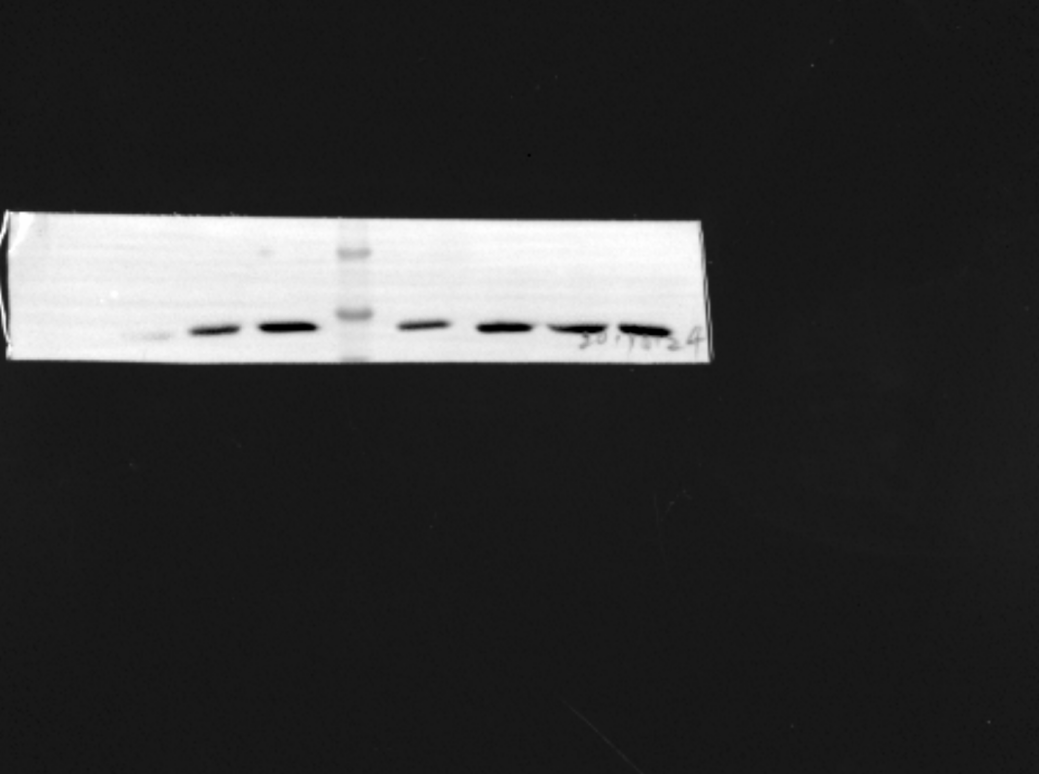


245kd

Fig4A JAG1 (left-BIU-87 right-MB49)


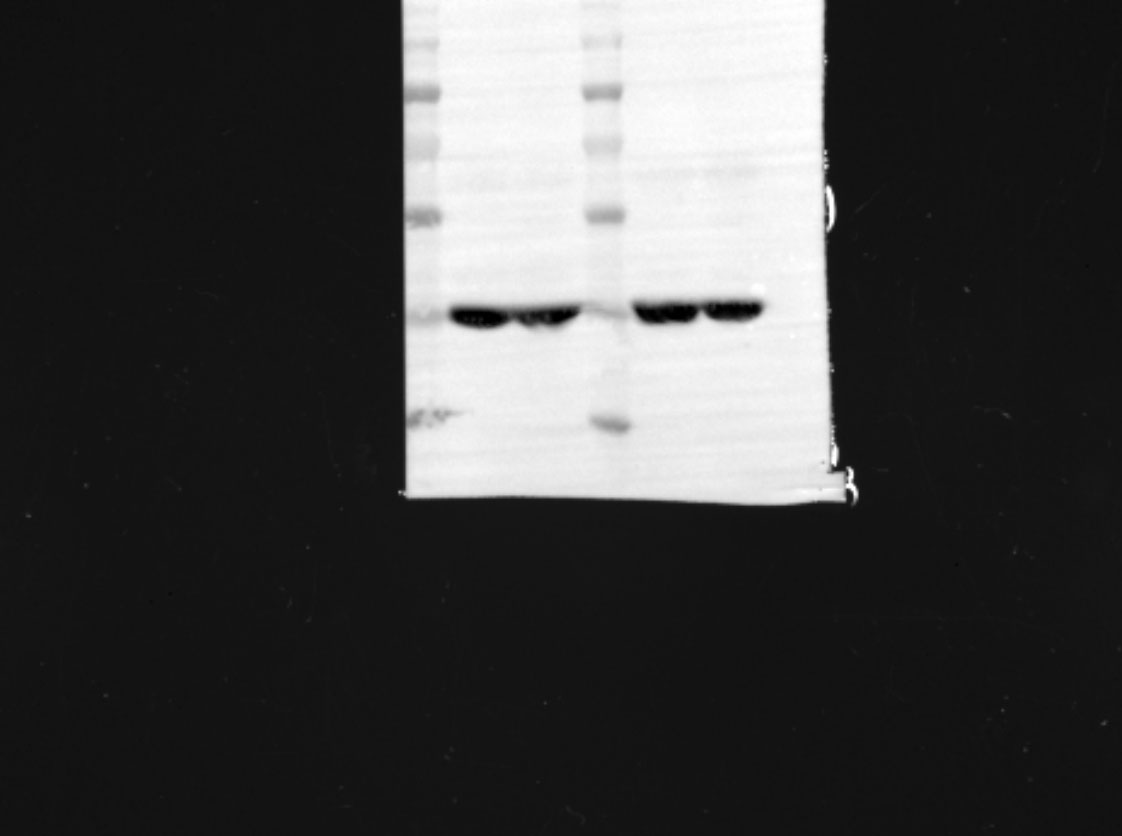


45kd

Fig4A Actin-2 (left-BIU-87 right-MB49)


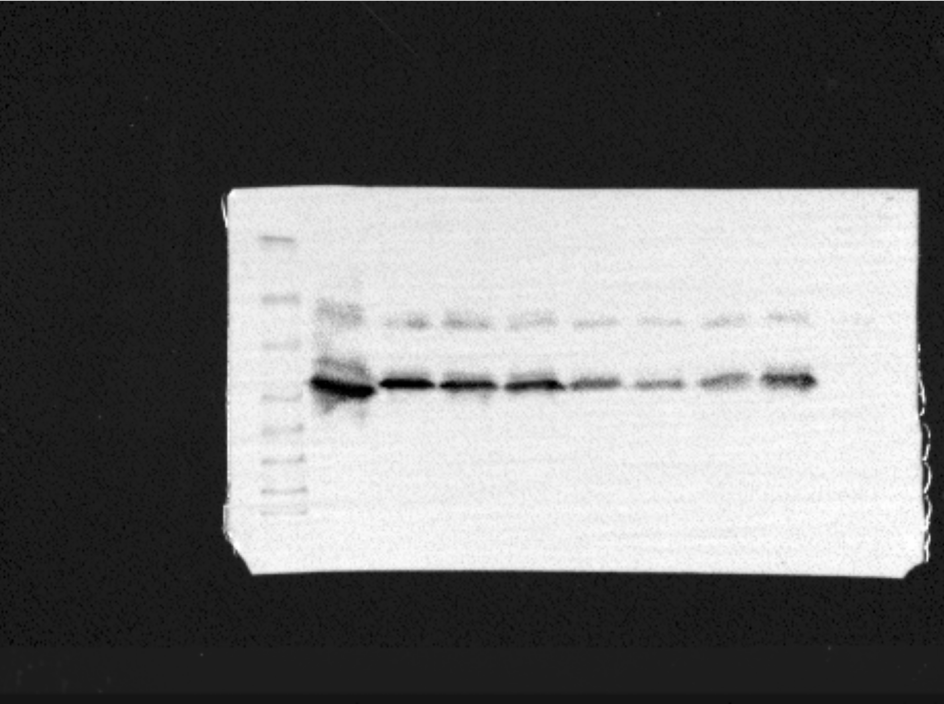


45kd

Fig4B TRB3


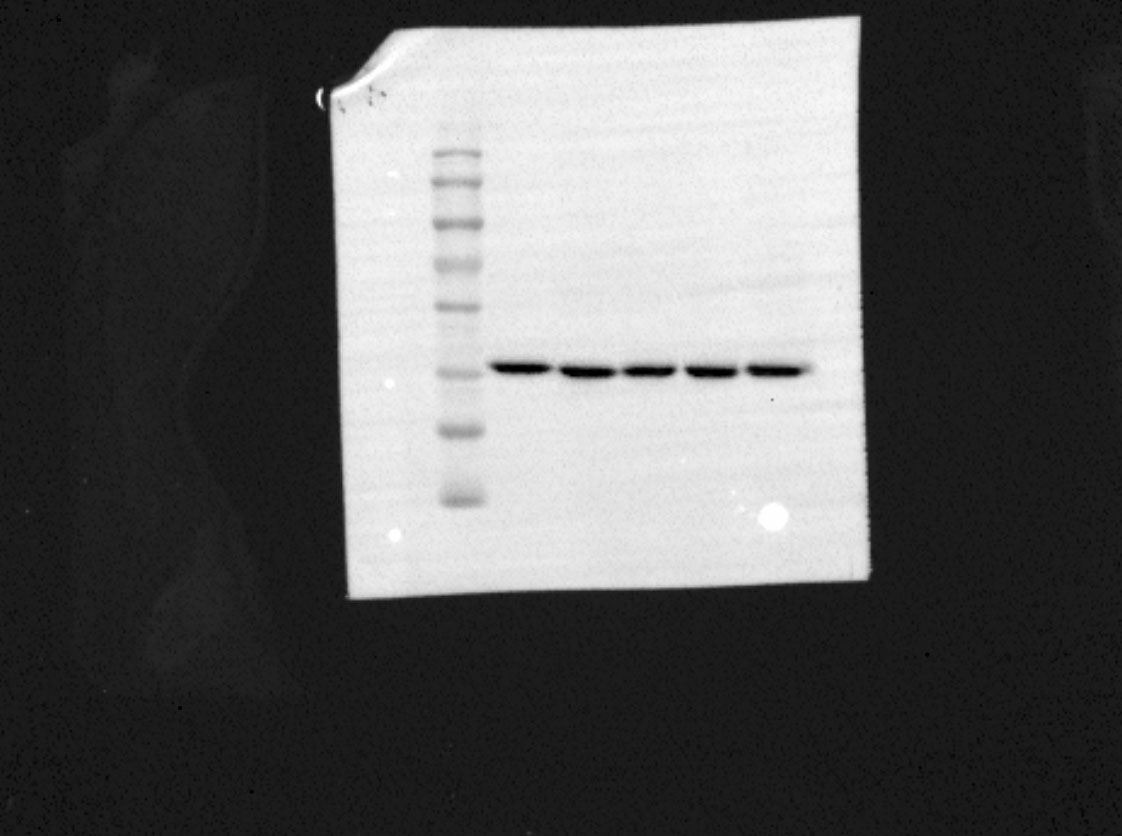


45kd

Fig4B Actin-1


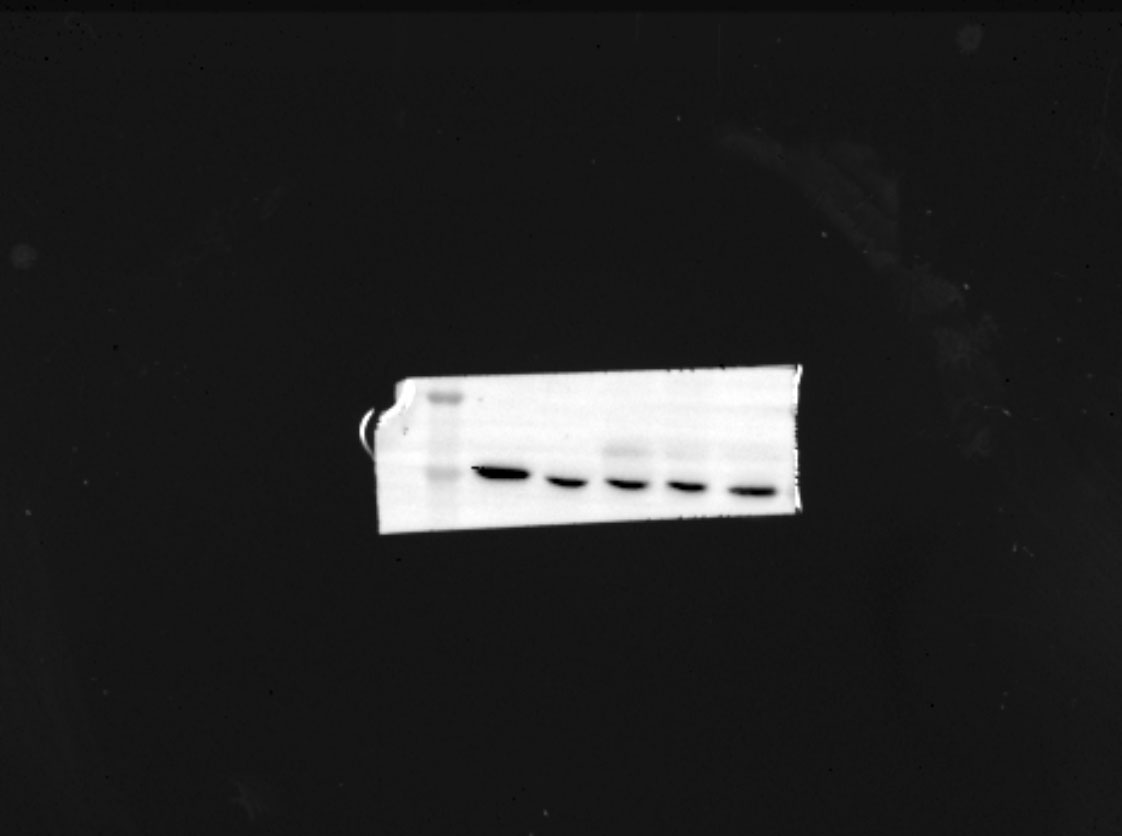


180kd

245kd

Fig4B JAG1


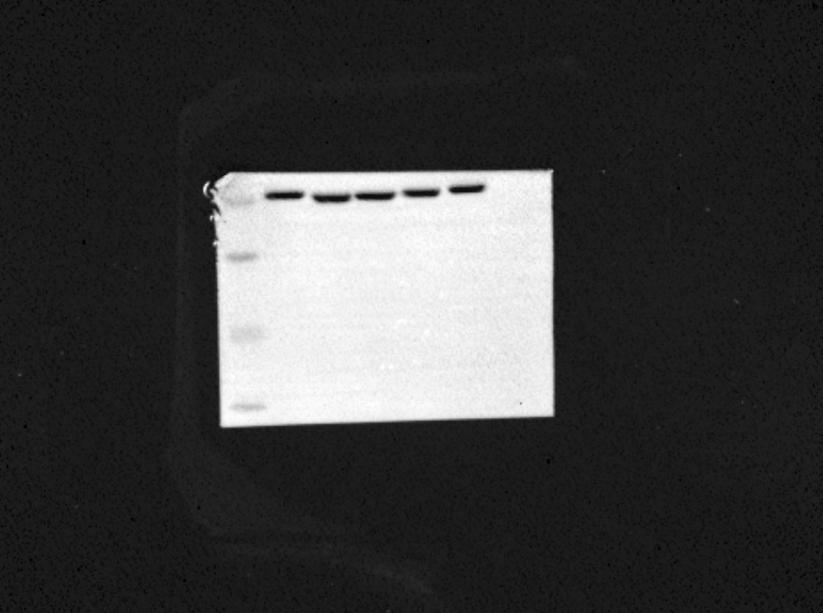


45kd

Fig4B Actin-2


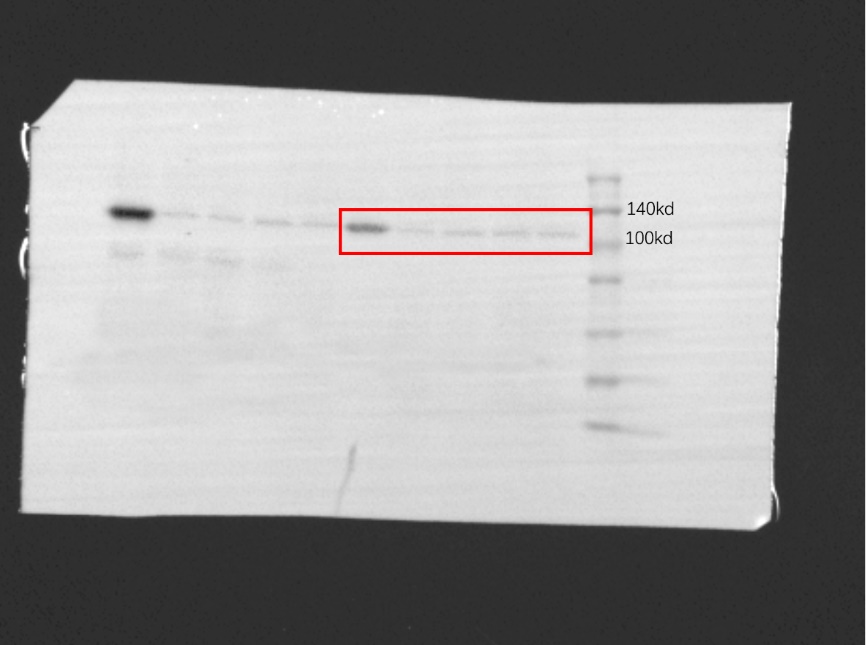


Fig4E Notch1


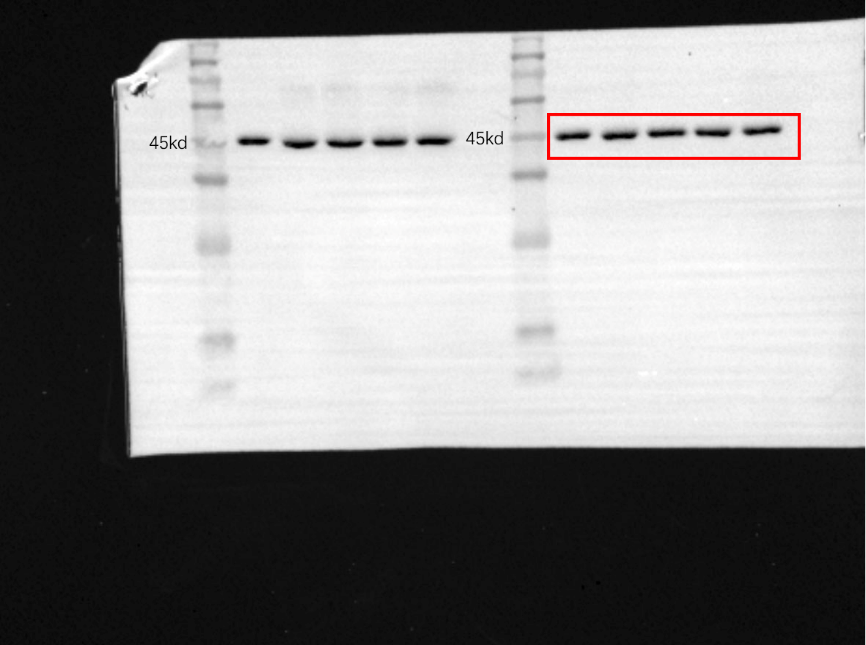


Fig4E Actin
